# Acute Pneumonia in Childhood*Read before the Hospital Graduates’ Club, November 23d, 1893.

**Published:** 1894-02

**Authors:** Thomas S. Southworth

**Affiliations:** New York; Pathologist to the Nursery and Child’s Hospital; Assistant Medical Department, Vanderbilt Clinic; Instructor in Diseases of Children, New York Polyclinic


### ACUTE PNEUMONIA IN CHILDHOOD.*

BY THOMAS S. SOUTHWORTH, M. D.,

Pathologist to the Nursery and Child's Hospital; Assistant Medical Department, Vanderbilt Clinic; Instructor in Diseases of Children, New York Polyclinic.

It has seemed to me that in an association of this nature it should be the endeavor of the members to present, when practicable, such subjects as shall touch upon the daily labor of the greatest numbers, rather than the curious and exceptional phases of medical experience. It is with this idea that I bring forward to-night the well-known subject of pneumonia in children, in the hope that each one whose general practice brings him into contact with children may contribute something to the discussion which will follow. As it is my aim, as far as possible, to avoid the hackneyed aspects of the subject, I will, without apology, make use of the terms lobar pneumonia and broncho-pneumonia as best expressing to me the essential differences of the two divisions of the subject. With the classical picture of lobar pneumonia—showing microscopically the pulmonary alveoli filled in varying degrees with fibrin, leucocytes, desquamated alveolar epithelium, and red-blood cells, but with an otherwise intact pulmonary framework—you are doubtless all familiar, as also with the four pathological stages of congestion, red hepatization, gray hepatization and resolution, through which the process passes.

Many of you, I presume, gained the impression from text-books that cases of lobar pneumonia were of extreme rarity under the fifth year. Statistics now show us that about thirty-three per cent. of the pneumonias of even the first two years of infancy show the well-marked clinical course and patholigical features of the lobar variety in the adult. These cases can usually with care be readily differentiated from those of broncho-pneumonia, although there are occasional instances over the classification of which the authorities are at variance.

Lobar pneumonia in a large proportion of cases is caused by an

*Read before the Hospital Graduates' Club, November 23d, 1893.

oval coccus called the *Micrococcus lanceolatus* (*Diplococcus pneumoniæ*, pneumococcus of Fraenkel). There are, however, occasional cases in which the clinical story and morphological character of the lesion are similar to those of lobar pneumonia, which are associated not with the *Diplococcus lanceolatus*, but with other germs, such as the bacillus of Friedländer, pyogenic bacteria, etc.*

Lobar pneumonia occurs most frequently during the spring months, and attacks by preference strong and previously healthy children, without especial preponderance of either sex. In the typical case the onset is sudden, ushered in most commonly by vomiting and a high temperature, occasionally by partial or general convulsions or a chill. The breathing is so rapid as to attract attention. The respiration has a characteristic rhythm; the quick, anxious inspiration is followed by a distinct pause before the explosive and often moaning expiration. Later the skin becomes dry and pale, and the cheeks flushed, the lips dry or cracked, but seldom presenting herpes. Cough, which may be painful and repressed, is of the hacking expiratory type. The pleural pain is often referred to the epigastrium, but may be correctly localized; and the child seeks a comfortable position, which it abandons unwillingly, even to receive fluids for the relief of thirst. The respirations are 40 to 60 per minute, the pulse 120 to 160 or even more, but regular, and the temperature varies from 102° to 105.5°, with slight morning remissions and evening rises. The development of the physical signs may be prompt or delayed. The respiratory murmur loses its vesicular character and becomes rougher, sharper, and of higher pitch until pure bronchial breathing is heard. Fine moist râles and rarely crepitant râles develop. The normal resonance is obscured and becomes marked dullness or dull tympany. The vocal resonance and fremitus is increased. These changes, once inaugurated, usually advance promptly until the consolidated area in one or more lobes can be clearly defined by physical exploration.

In simple and favorable cases the crisis occurs between the fifth and ninth days, occasionally between the third and fifth days. The temperature in twelve to twenty-four hours drops several degrees to below the normal, with sweating and somnolence, and the child falls

*The question whether a more exact distinction between the various forms of exudative pneumonia, based primarily on the germs causing them, is not desirable, is one which does not fall within the scope of this paper.

asleep to awake greatly improved. The change in the appearance of the patient in a few hours is astonishing—faciës, pulse, respiration, appetite and secretion are all changed for the better; but the signs of consolidation and the cough remain at first unmodified, to disappear with inexplicable rapidity during the succeeding days.

Did all cases follow this classical course there would be few difficulties in the way of early and complete diagnosis; but there are many anomalies, and it is these variations from the normal which cause us the greatest anxiety, and, theretore are of the greatest interest. They may be grouped as†—

1. Abortive pneumonia.
2. Wandering pneumonia.
3. Gastric pneumonia.
4. Cerebral pneumonia.

The *abortive cases* begin with all the rational signs of pneumonia. and the diagnosis seems clear, although examination may reveal only modified resonance or slight dullness, rough or broncho-vesicular breathing or localized râles, when, to our complete surprise, in twenty-four to thirty-six hours there is a sudden disappearance of both physical signs and symptoms, with speedy convalescence. These are undoubtedly instances of localized hyperemia of the lung tissues which, for some unexplained reason, do not go on to complete consolidation.

Far too little attention has been given in the text-books to these areas of congestion in the lung which, existing alone, constitute the abortive form of the disease, or which may precede or accompany the development of true pulmonary consolidation. The omission has led to much confusion and lack of self-confidence among those who, seeing children but little, are mortified and disconcerted, to find signs which they had one day satisfactorily established, the next day completely absent. These fleeting patches, for whose erratic development and localization we can find no satisfactory explanation, are not confined to the lobar form, but play, as we shall see, a very important part in the course of broncho-pneumonia. The congestion and engorgement of the pulmonary tissue give rise to slight or more marked dullness, to distant or distinct bronchial breathing according to their intensity. Pulmonary relaxation may add a tympanitic quality to the percussion note. Obstruction to the entrance ot air may result in the decreased respiratory murmur, while the penetration of air into the partly occluded vesicles accounts for the scattered râles.—*N. Y. Med. Jour. January 3, 1894.*

†Baginsky. *Praktische Baiträge zur Kinderheilkunde*, 1830.

(*To be continued.*)

